# A Familial Case of Robertsonian Translocation 13;14: Case Report

**DOI:** 10.7759/cureus.29430

**Published:** 2022-09-21

**Authors:** Sondas Saeed, Jawad Hassan, Sarah M Javed, Saira Shan, Maliha Naz

**Affiliations:** 1 Cytogenetics, National Institute of Blood Disease and Bone Marrow Transplantation, Karachi, PAK; 2 Haematology, National Institute of Blood Disease and Bone Marrow Transplantation, Karachi, PAK

**Keywords:** genetic counseling, cytogenetic analysis, primary amenorrhea, acrocentric, robertsonian translation

## Abstract

Robertsonian translocations are the most common form of chromosomal abnormalities that specifically involve the acrocentric chromosomes. Robertsonian translocation between chromosomes 13,14 and 14,21 are the most frequently reported. Infertility is common in genetically balanced carriers of these translocations, and their conceptions are more likely to have imbalances. Here we have reported a case of an 18-year-old female presenting with a complaint of primary amenorrhea. Cytogenetic analysis revealed a familial case of maternally inherited Robertsonian translocation (rob(13;14)(q10;q10)) affecting all the siblings. Genetic counseling and genetic testing are recommended especially in familial cases as the carriers are normal but can lead to several genetic disorders in their future generation.

## Introduction

Robertsonian (rob) translocation is the most common form of structural chromosomal abnormality or rearrangement [1-3]. One in 1,000 healthy individuals is thought to carry a Robertsonian translocation inherited from one of the parents with a normal phenotype [2-4]. A Robertsonian translocation involves only the acrocentric chromosomes (13, 14, 15, 21, and 22). It results from the breakage of two acrocentric chromosomes at or close to their centromeres, with a subsequent fusion of the long arms to form one metacentric chromosome. The individual who has a balanced translocation is referred to as a Robertsonian translocation carrier. Many carriers never learn about their chromosomal rearrangement since carriers typically exhibit no negative health consequences or reduced life span. The translocation can thus be passed through several generations without detection. The most common form of Robertsonian translocations is between chromosomes 13 and 14, which accounts for approximately 75% of all translocations [2,5]. Carriers of rob(13;14) are phenotypically normal but there is an increased risk that they can produce unbalanced gametes that will result in monosomic or trisomic fetuses. Some carriers can also face problems related to reduced fertility, unfavorable pregnancy outcomes like spontaneous abortions, stillbirths, offspring with intellectual disabilities, and uniparental disomy-related complications [4,6,7].

Here we report a familial case of Robertsonian translocation, in which the proband was referred because of primary amenorrhea.

## Case presentation

An 18-year-old female presented with a history of primary amenorrhea. Pelvic ultrasound revealed incomplete development of Mullerian ducts, a thin elongated uterus unicornis unicollis was present on the left side, while a small rudimentary horn of uterus was present on the right side measuring 7.0 x 0.8 cm, having normal ovaries. She was referred to the National Institute of Blood Disease & Bone Marrow Transplantation for cytogenetic study.

A peripheral blood sample was received in a sodium heparin tube and was processed for 72 hours in phytohemagglutinin (PHA) stimulated cultures as per standard protocol. After 72 hours, cells were harvested and standard G-banding protocol was carried out to prepare slides for chromosomal analysis. When 25 metaphase spreads were observed, counted, and karyotyping was done with 400 G-banding resolution using Ikaros karyotyping system-Metasystems software (Carl Zeiss Microscopy Gmbh, Göttingen, Germany). Analysis was done according to the guidelines provided by International System for Human Cytogenetic Nomenclature (ISCN).

**Figure 1:**
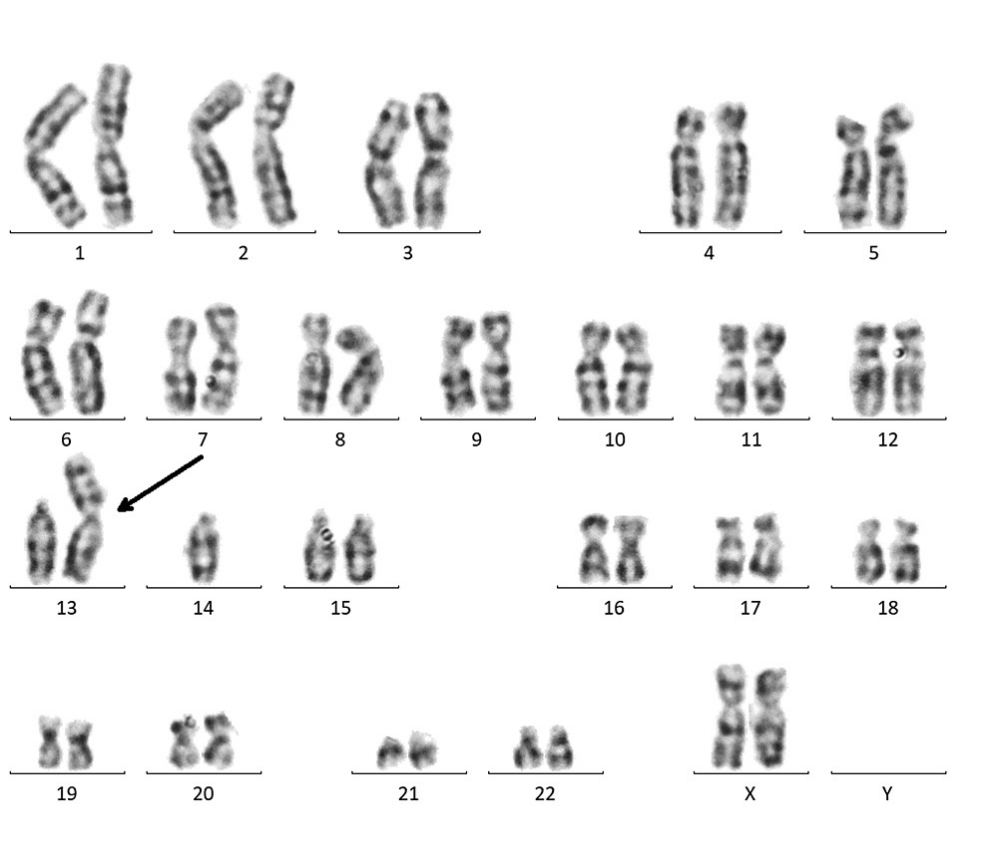
Proband karyogram. Karyogram of proband showing Robertsonian translocation (45,XX,rob(13;14)(q10;q10)).

In the proband, Robertsonian translocation between chromosomes 13 and 14 was detected in all observed metaphases (45,XX,rob(13;14)(q10; q10)) (Figure 1).

**Figure 2:**
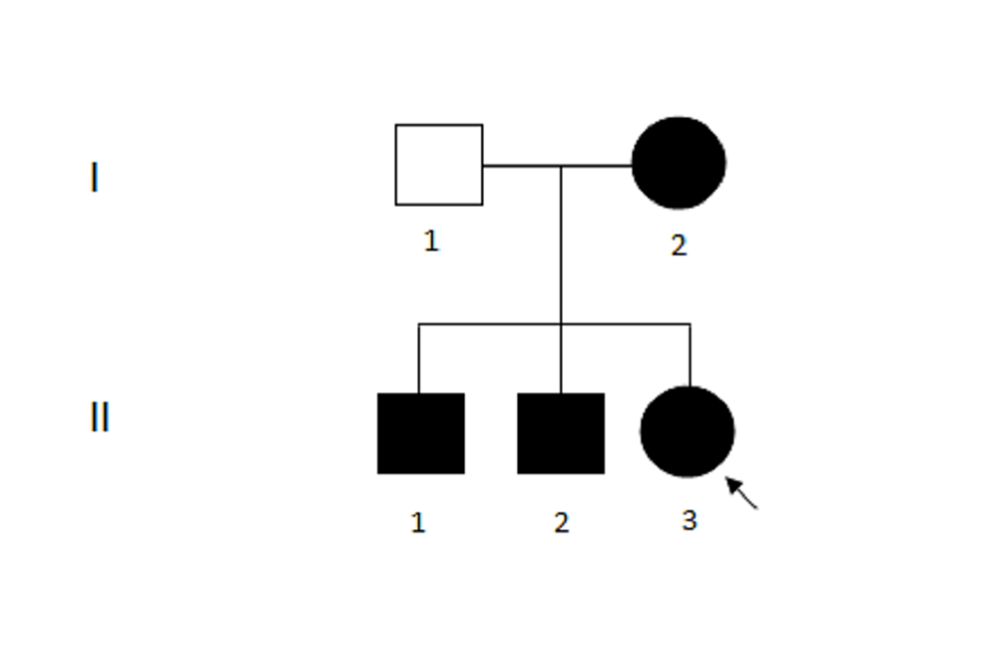
Family pedigree. Pedigree representing mother (I-2), proband (II-3) with karyotype (45,XX,rob(13;14)(q10;q10)) and brothers (II-1, II-2) with karyotype (45,XY,rob(13;14)(q10;q10)) as dark circles and squares respectively. Father (I-1) with normal karyotype 46,XY is shown as an unfilled square.

To ascertain the origin of the Robertsonian translocation in the proband, cytogenetic analyses of first-degree family members were also performed. Family members include a father, a mother, and two elder brothers (Figure 2). No history of miscarriages or stillbirth was reported in the proband’s family.

**Figure 3:**
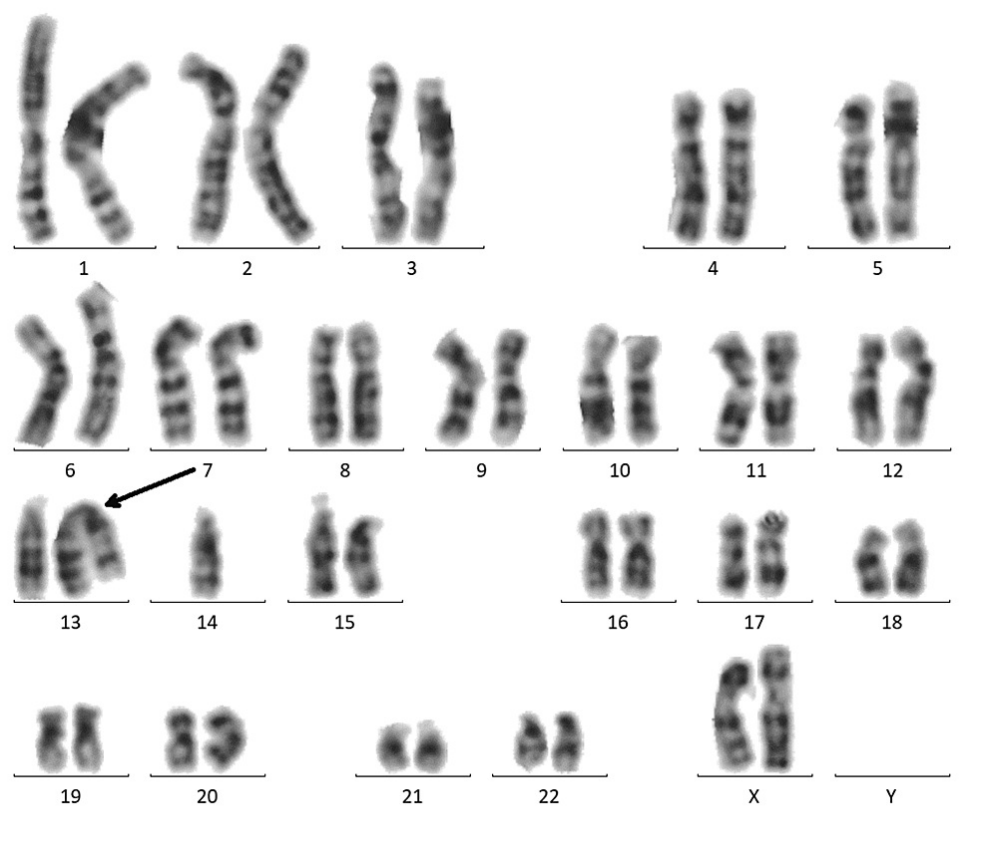
Mother's karyogram. Karyogram of mother showing Robertsonian translocation (45,XX,rob(13;14)(q10;q10)).

**Figure 4:**
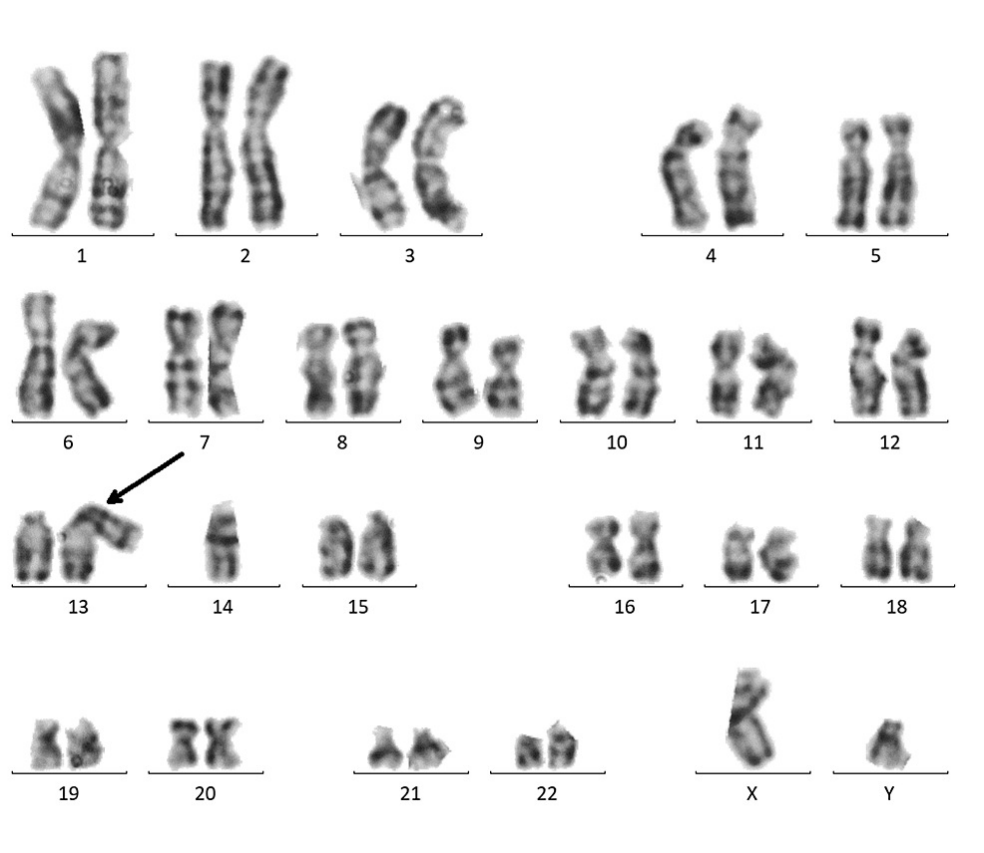
Brother's (II-1) karyogram. Karyogram of the brother (II-1) showing Robertsonian translocation (45,XX,rob(13;14)(q10;q10)).

The chromosomal analysis of the proband’s father revealed a normal 46,XY karyotype (not shown), whereas the mother and brothers carried the same Robertsonian translocation (rob(13;14)(q10; q10)). The karyogram of the proband’s mother is shown in Figure 3 and the karyogram of one brother (II-1) is shown in Figure 4. The other brother exhibited the same Robertsonian translocation (not shown).

 

## Discussion

A female heterozygous carrier of Robertsonian translocation (13q;14q) was reported in this paper and presented with primary amenorrhea along with a rudimentary uterus. The proband was born to non-consanguineous parents; cytogenetic analysis confirmed the mother is the carrier of the Robertsonian translocation though no adverse pregnancy outcome was reported in the parents. Cytogenetic analysis of this family revealed a strong familial case of Robertsonian translocation (rob(13;14)(q10;q10)), in which the father exhibited normal male karyotype (46,XY), whereas all the siblings carried the same abnormality inherited from the maternal side.

An individual with a balanced Robertsonian translocation and a normal phenotype has a 50% chance of passing on the translocated chromosome to each gamete produced. Statistically, 2/3 of the gametes produced by an individual with a balanced Robertsonian translocation will have unbalanced chromosomes, and when these gametes are fertilized, the conceptuses they produce will have unbalanced karyotypes. Trisomy of chromosome 13 is almost usually fatal during pregnancy or soon after birth, whereas monosomy of chromosomes 13 or 14, or trisomy of chromosome 14, causes fetal death [8]. As per the probability of inheritance, 50% of all viable offspring will have a balanced translocation. Thus, there is a slightly less than 1/8 chance of the pedigree in this report being produced simply by random chance.

Such a strong inheritance suggests that it might be a case of uniparental disomy (UPD). Rob(13;14) carriers, like all rob carriers, are more likely to develop UPD [9]. Numerous studies revealed that a Robertsonian translocation involving chromosome 14 was correlated to UPD [10,11]. A cytogenetically verified or suspected normal karyotype of 46,XX or 46,XY is present in 65% of the documented UPD cases. However, cytogenetic testing isn’t performed in more than 50% of these instances which is particularly important in cases of UPD involving chromosomes 13, 14, 15, 21, or 22. This uncommon type of mitotic error is known to arise in the presence of a Robertsonian translocation [12]. UPD is reported in 0.6-3% of cases having Robertsonian translocations [13]. However, Robertsonian translocation is present in more than 10% of the reported UPDs produced from acrocentric chromosomes [14].

Genetic counseling is crucial for carriers, particularly in familial situations as the carriers of a balanced (rob(13;14)) are normal and any clinical heterogeneity resulting from the (rob(13;14)) may be caused by various genetic imbalances. By carefully mapping the breakpoints involved in these translocations, a more precise definition of this clinical characteristic heterogeneity would be possible.

## Conclusions

We have reported a familial case of Robertsonian translocation involving chromosomes 13 and 14 in which all of the offspring were affected. Such strong inheritance can be a case of maternal UPD but requires further molecular testing for confirmation. The association of Robertsonian translocation with primary amenorrhea along with the rudimentary uterus may be coincidental and hasn't been reported yet.
